# A de novo variant of CHD8 in a patient with autism spectrum disorder

**DOI:** 10.15190/d.2020.4

**Published:** 2020-03-31

**Authors:** Maha Alotaibi, Khushnooda Ramzan

**Affiliations:** Department of Genetics, King Saud Medical City, Riyadh, Saudi Arabia; Department of Genetics, King Faisal Specialist Hospital and Research Centre, Riyadh, Saudi Arabia

**Keywords:** Autism spectrum disorder, macrocephaly, nextgeneration sequencing, de novo, CHD8 mutation, Saudi Arabia.

## Abstract

Autism spectrum disorder (ASD) is a heterogeneous group of neurodevelopmental disorders, usually diagnosed in early childhood, that are characterized by adaptive deficits in social interaction, communication skills, and restricted or stereotyped repetitive patterns of behavior. There had been limited success to define ASD subtypes on the behavioral basis. Genetically categorized ASD subtypes may provide basis to determine the course, prognosis, and individualized mechanism based treatment. Mutations in chromodomain helicase DNA-binding protein 8 (CHD8) gene, have been associated with autism, macrocephaly, speech delay, distinct facial features, sleep and gastrointestinal disturbances. There are few cases in the literature reporting de novo mutations of CHD8 exhibiting sporadic ASD. Here we describe a Saudi boy with developmental delay, intellectual disability, macrocephaly, craniofacial abnormalities, speech delay, but without any history of seizures, gastrointestinal problems or sleep disturbance. Whole exome sequencing for parent-child trio revealed a de novo heterozygous loss-of-function mutation (c.4984C>T, p.Arg1662Ter) in CHD8 gene. Our findings elaborate the genotype-phenotype correlation and confirm that the CHD8 disruptions represent a clinical ASD subtype and further highlight the significance of implementing genomic medicine in clinical practice for an early intervention and necessary support for the families.

## INTRODUCTION 

Autism spectrum disorder (ASD) encompasses a group of clinically heterogeneous neurodevelopmental disorders, that are characterized by impaired social communication, language deficits, restrictive interests or stereotyped repetitive behaviors^1,2^. The estimated prevalence of ASD is about 0.12-2.64% in different populations^3^. In Saudi Arabia, the prevalence of ASD is about one per 167 (0.6%), suggesting the total number of individuals with ASD is over 167,000 in a population of over 28 million people^4^. There is no available comprehensive data detailing the confirmed cases of ASD in the Saudi population. ASD has significant genotypic and phenotypic complexity with diverse genetic and non-genetic etiologies. Molecular studies have identified more than 100 ASD-risk genes and genomic regions associated with the pathogenesis of ASD. However, for at least 70% of the cases, the underlying genetic cause remains unidentified^5,6^. With the advent of next generation sequencing technologies, mutations in various genes underlying the phenotype of ASD have been discovered. Mutations in the chromodomain helicase DNA-binding protein 8 (CHD8) gene are associated with the classic form of autism, together with macrocephaly, distinct dysmorphic facial features, and gastrointestinal disturbance^7^. 

In this article, we report a case of a Saudi child with intellectual disability and autistic behaviour, diagnosed by the clinical examinations and confirmed by genetic testing, together with whole exome sequencing (WES), which led us to the identification of a de novo mutation of CHD8 gene.

## CASE REPORT 

A 7-years-old Saudi boy was referred to the genetic clinic with the history of developmental delay. He sat at the age of one year and walked after two and a half years. He was the child of healthy Saudi consanguineous parents with an older sibling, she was a 9-years-old healthy girl with good school performance. The mother did not have any history of abortions. Pregnancy and delivery were normal. He was born at full term with normal measurements (birth weight: 3.2kg, on 5th percentile). Examinations at the age of 6 years old was notable for dysmorphic facial features with a large head, pronounced supraorbital ridge with down-slanting palpebral fissures, broad nose, and smooth philtrum. The head circumference was in the 90th percentile, while the height and weight were on the 5th centile. The affected boy had significant speech delay. He was not interested in his surroundings and had an abnormal social interaction with an aggressive behaviour. He showed poor eye contact and stereotypic, repetitive behaviours such as putting things in the same spots and failed to finish any of the activities. According to his parents, he does not show any improvement in his performance in school. No formal IQ assessment was done. There was no history of any such similar condition or psychiatry illness in the family. Magnetic resonance imaging (MRI) of the brain was unremarkable, and there was no history of seizures. Ophthalmological and hearing assessment were normal.

## GENETIC STUDIES 

Genetic analyses were performed after obtaining a signed informed consent from the patients’ parent. Chromosome analysis revealed a normal male karyotype (46, XY) and the fragile X test was unremarkable. Single nucleotide polymorphism (SNP) array did not detected any definite pathogenic copy number variant (CNV). WES was performed using the Agilent SureSelect Target Enrichment workflow and the Illumina HiSeq 2500 sequencing system. The obtained exome DNA sequences were mapped and compared to the human genome build hg19 reference sequence. Exome analyses interrogated thousands of genetic variants in a proband using proprietary database softwares and pipelines customized to Arab populations. The identifed variants are characterized by using the American College of Medical Genetics and Genomics (ACMG) guidelines to classify their clinical relevance^8^. 

A heterozygous CHD8 (NM_0011770629) variant in exon 26 (c.4984C>T; p.Arg1662Ter) was identified in the patient by WES. Sanger sequencing was performed to confirm the validity. WES for the parents was unremarkable, which indicated the de novo status of the identified CHD8 variant in the affected boy. The identified variant was not found in ExAC or 1000G population database. The variant creates a premature stop codon (p.Arg1662Ter) leading to the loss of 919 amino acids of the protein and was determined to be pathogenic according to ACMG guidelines. 

## DISCUSSION 

The CHD8 gene located on 14q11.2 encodes the CHD8 protein, which has been associated with ASD by independent studies. CHD8 protein acts as a regulator in the Wnt/β-catenin signaling pathway, that binds directly to β-catenin and suppresses its transactivation activity and is a potential regulator of WNT signaling which has a role in vertebrate development and morphogenesis^9-11^. CHD8 interacts and regulates the co-expression of other ASD-risk genes during the human brain development^12^. Noteworthy is its interaction with CHD7, a gene mutated in CHARGE syndrome, which involves a clustering of congenital anomalies affecting several tissues^13,14^. CHD8 also has a essential role in activation of transcription factors regulating cell cycle progression and maturation^15^. 

CHD8 disruptions thereby present a specific pathway in the ASD development due to the abnormal cortical development, therefore presenting a distinct constellation of symptoms for this ASD-subtype. 

Animal studies in both zebrafish and mouse models recapitulate phenotypic features of disruptive mutations of CHD8 in humans. Mice with Chd8 mutations have macrocephaly, craniofacial abnormalities, learning and memory defects and autism like phenotype^7,16^. The chd8 disruption in zebrafish leads to an increased head size due to expansion of the forebrain/midbrain and to problems in gastrointestinal motility. CHD8 disruptions thereby present a specific pathway in the ASD development due to abnormal cortical development, therefore presenting a distinct constellation of symptoms for this ASD-subtype. Patients with CHD8 mutations have variable presentation and in addition to autistic behaviors, other phenotypic features also co-exist including macrocephaly, rapid postnatal growth; distinct faces (prominent forehead, wide set eyes, pointed chin), gastrointestinal and sleep dysfunctions. The macrocephaly, facial features and gastrointestinal complaints are most notable when compared to other ASD patients without CHD8 mutations^7,17^. 

A variety of CHD8 genotypes have been reported by whole-genome copy number variation (CNV) studies, next-generation sequencing (NGS) technologies and candidate re-sequencing studies. Chromosomal microdeletions, including a recurrent ~ 100 Kb microdeletion, balanced chromosomal abnormalities, and de novo missense and nonsense mutations, have been involved in the pathogenesis of the ASD^7,18-21^. 

The mutations of CHD8 gene have significant role in ASD as studied in two unrelated children with developmental delay, and normal brain as shown using MRI. Both were dysmorphic (short palpebral fissures and long philtrum), while one of them had large head and the other had normal head circumference^22^. In another study fom China, three patients with de novo mutation were reported. Two of them had similar phenotype with intellectual disability, macrocephaly, anxiety, sleep and gastrointestinal disturbances^23^. CHD8 was sequenced in a large cohort of 3,730 children with ASD. The patients bearing CHD8 mutations had distinctive characteristics including distinct facial features, macrocephaly, and gastrointestinal complaints^7^. There are no molecular studied reported cases of CHD8 mutations in the Saudi population. We identified a novel de novo loss-of-function CHD8 mutation in a Saudi child diagnosed with ASD and developmental delay. He had similar clinical characteristics consistent with previously reported patients with CHD8 mutations, including the macrocephaly and craniofacial abnormalities, although, for our patient there were no complaints of gastrointestinal or sleep disturbances. 

CHD8 mutations have been previously associated with ASD schizophrenia in adults and different gastric or colorectal cancers, which indicates its relation to schizophrenia and autism at the gene level^24^. Clinicians should pay attention to what would be expected in the future regarding the the patients with CHD8 mutations and their family members. The loss of CHD8 expression could be a novel indicator for these disorders and CHD8 ASD patient should be screened for early signs of gastric or colorectal cancer, schizophrenia or other related psychosis disorder, such as depression and anxiety^25,26^. 

## CONCLUSION 

In conclusion, we identified a de novo mutation of CHD8 gene in a Saudi child, with a phenotypically heterogeneous ASD-subtype. Mutations associated with ASD further provide a rich source for understanding the involved pathogenic genes and neurobiological pathogenesis of ASD. From the clinical perspective, genetically-based ASD subtype classification helps in early diagnosis, broader our understanding of the course and long-term prognosis, and mechanisms-based tailored treatments for a sub-group of patients^27^. In addition, this is also important for the clinical evaluation and family counseling.
